# Optimization of Gas Sensors Based on Advanced Nanomaterials through Split-Plot Designs and GLMMs

**DOI:** 10.3390/s18113858

**Published:** 2018-11-09

**Authors:** Rossella Berni, Francesco Bertocci

**Affiliations:** 1 Department of Statistics Computer Science Applications “Giuseppe Parenti”, University of Florence, Viale Morgagni 59, 50134 Florence, Italy; 2 Department of Information Engineering and Mathematics, University of Siena, Via Roma 56, 53100 Siena, Italy; francesco.bertocci@live.com

**Keywords:** experimental design, Generalized Linear Mixed Models, metal oxide semiconductors, resistive gas sensors, robust process optimization

## Abstract

This paper deals with the planning and modeling of a split-plot experiment to improve novel gas sensing materials based on Perovskite, a nano-structured, semi-conductor material that is sensitive to changes in the concentration of hazardous gas in the ambient air. The study addresses both applied and theoretical issues. More precisely, it focuses on (i) the detection of harmful gases, e.g., NO${}_{2}$ and CO, which have a great impact on industrial applications as well as a significantly harmful impact on human health; (ii) the planning and modeling of a split-plot design for the two target gases by applying a dual-response modeling approach in which two models, e.g., location and dispersion models, are estimated; and (iii) a robust process optimization conducted in the final modeling step for each target gas and for each gas sensing material, conditioned to the minimization of the working temperature. The dual-response modeling allows us to achieve satisfactory estimates for the process variables and, at the same time, good diagnostic valuations. Optimal solutions are obtained for each gas sensing material while also improving the results achieved from previous studies.

## 1. Introduction

This paper illustrates a case study involving a split-plot design and modeling. The study is related to the analysis of experimental data for eight different gas sensing materials based on metal oxide semiconductors (MOX), e.g., Perovskites. Perovskites, consisting of YCoO${}_{3}$-based powder, have been developed for the detection of harmful gases and for industrial applications. These materials have received considerable attention in recent years [1,2], due to their ability to detect chemical contaminants in the ambient air, including the byproducts of combustion, such as nitrogen dioxide, NO${}_{2}$, and carbon monoxide, CO, which are known as target gases (gas). The latter are extremely toxic, even at low concentrations, and in urban areas they can reach high concentrations, especially near busy roads or industrial areas. Indeed, nitrogen dioxide is an important air pollutant because it contributes to the formation of photochemical smog which has a very harmful impact on human health.

The case study is carried out by considering a split-plot design [3], and a dual-response modeling approach, where the location (main) response model related to the response variable of the studied technological process is weighted to take overdispersion into account. Furthermore, a dispersion model is jointly fitted, and the weights for the main model are estimated; weights are included in the estimation of the location model for evaluating overdispersion. Random and fixed effects are opportunely involved in the design planning and modeling in order to achieve statistical results as accurate as possible and also to comply with the operative and functional characteristics of the process under examination for the purpose of providing further confirmation of the dual-response modeling approach. Optimization is performed by satisfying stringent technological requirements and achieving the best experimental combination for the detection of harmful gases, e.g., NO${}_{2}$ and CO. Moreover, the process of optimization establishes a solid basis for the application of sensors in the industrial sector and for environmental pollution monitoring. In addition, the research is a further improvement on two previous studies [4,5], in which gas sensing materials were studied through mixed response surface models. In [4], the application was used on observational data, e.g., without planning an ad-hoc experimental design, while in [5], a split-plot design was planned and modeled through mixed response surface models. Nevertheless, in [5], the optimization was carried out through an analytical approach that allowed steady and valid model results to be obtained, even though some of these were not completely satisfactory in engineering terms due to the small number of observations for each sensing film. In what follows, we plan to show the improvements achieved through this new study by considering the following three innovative contributions:

- A split-plot design, modified with respect to the split-plot applied in [5] with N=96 observations is planned, as detailed in Section 4. The data are then modeled through a quasi-likelihood, dual-response approach using Generalized Linear Mixed Models (GLMMs), which allows for better modeling of environmental (noise) effects and overdispersion; a specific structure, e.g., a compound symmetry structure is evaluated for variances and covariances of the random effects;
- The dual-response approach is carried out through two GLMMs—one for location effects and the other for dispersion effects—by simultaneously including both target gases, i.e., NO${}_{2}$ and CO. One of the reasons for performing a dual approach for studying both gases is the need to weight the location model for overdispersion and to achieve the highest values for the response, also obtaining a better goodness-of-fit of the location model;
- The optimization is carried out through the estimated location model, e.g., without applying an analytical procedure, and the optimal values for gas sensing materials are calculated by estimating the predicted values.

The paper is organized as follows: Section 2 includes a description of the case study, particularly focusing on the sensor structure and the system process; Section 3 contains an explanation of the basic theory related to the GLMMs and the split-plot design and includes the applied dual-response modeling approach; Section 4 describes the planning of the split-plot design; Section 5 illustrates the case study results, e.g., the split-plot modeling results in a GLMM context, also including diagnostic evidence; Section 6 contains a discussion on the optimized sensor material values obtained, followed by concluding remarks.

## 2. The Case Study

The possibility of designing gas sensors with advanced materials able to combine the typical advantages of this sensing technology, i.e., large sensitivity and low cost, with enhanced stability and reliability over time is stimulating ongoing research on Perovskite metal oxides [6].

Perovskite, $YCoO_{3}$, is a nano-structured, semi-conductor material that is sensitive to changes in the concentration of hazardous gas in the ambient air. The variations in electrical properties of perovskite are due to the the presence of NO${}_{2}$ and CO in the surrounding atmosphere, and as a result, the pollution degree and toxic gas concentrations become measurable.

It should be underlined that it is very difficult to predict the potential behavior of materials used as gas sensors. The MOX gas sensor response is a complex function of many quantities and parameters which makes it necessary to test the new materials in different environments and at different temperatures [7,8]. Due to the burden of obtaining responses from these devices, the required measurement campaigns represent a significant outlay in terms of time and budget [9].

The variation in the electrical resistance of the gas sensing film is the sensor output, also called the response, and is denoted by *Y* in the statistical analysis. For this study, the sensor response is defined as follows:

(1) $$Response\left[\%\right]=\frac{\left(R^{v}-R_{0}^{v}\right)}{R_{0}^{v}}*100.$$

In Equation (1), $R_{0}^{v}$ is the baseline resistance value obtained at the considered temperature in a reference gas (air), whereas $R^{v}$ is the resistance value after a fixed duration exposure to a target gas at a given concentration.

**Figure 1:**
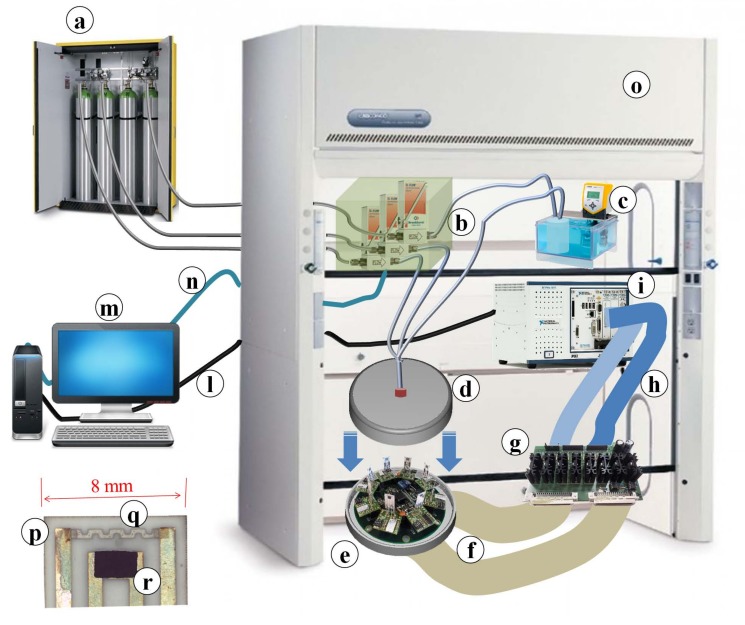
(**a**) Gas reservoir; (**b**) mass flowmeters; (**c**) bubbler; (**d**) stainless steel measurement chamber; (**e**) array of eight sensors; (**f**,**h**) signal connection cables; (**g**) heater drivers; (**i**) NI-PXI (PXI System-National Instruments) for real-time application; (**l**) ethernet link; (**m**) host computer with NI Labview Software for automated tests; (**n**) serial link; (**o**) extractor hood; (**p**) alumina substrate; (**q**) temperature sensor; (**r**) screen-printed sensing material.

The experimental data presented in this work were obtained by means of the high accuracy measurement system described in Figure 1 (see also [10]) that was developed to simultaneously characterize up to eight sensors mounted in a circular array (Figure 1 part e) inside a stainless steel chamber (Figure 1 part d). The measurement system was designed to study the behavior of the sensors by accurately setting the operating conditions in terms of the chemical environment composition (Figure 1 part a), gas flow (Figure 1 part b), humidity (Figure 1 part c) and temperature (Figure 1 parts f, g). The system is fully programmable and it individually controls the working temperatures of the sensors (Figure 1 parts h, i, l–n). These features make the system suitable for determining the principal performance indexes of a gas-sensing device (i.e., response—Formula (1) ) as functions of various combinations of measurement conditions (e.g., gas concentrations, temperature, humidity, and flow) [11]. The sensing film can be realized on an alumina substrate (Figure 1 part p) equipped with electrodes in order to measure the electrical resistance, while a heater and an accurate temperature sensor provide the working conditions (Figure 1 part q). The material (sensing film) studied (Figure 1 part r) was deposited on this structure using the screen printing technique. The whole measurement system was contained in an extractor hood (Figure 1 part o) in order to protect the research laboratory from toxic gases.

Eight gas sensing materials were studied (Table 1). The base material was Mt5 which was proposed and studied as a gas sensing material both for carbon monoxide and nitrogen dioxide gases [6]. Mt1, Mt2, and Mt3 are variants of the base material that were obtained by inserting palladium, (Pd), because they show better gas sensitivities, especially for NO${}_{2}$ (see [4]). Mt4 was obtained by adding a major molar volume of Yttrium (Y) during the preparation; Mt6 was obtained by removing a few molar volumes of cobalt (Co) (see also [2]). Mt7 and Mt8 were prepared by using the impregnation technique and adding 1% Pd on the nano-particle surfaces of Mt5 and Mt6 respectively [6]. A furnace was used for the heat treatment of all these materials.

## 3. Theory

In this section, we briefly describe some fundamental elements of the theoretical issues related to GLMMs as well as split-plot design and modeling. The GLMMs are briefly illustrated in Section 3.1, split-plot design and modeling are illustrated in Section 3.2, and the suggested dual-response modeling approach is presented in Section 3.3. For further detail, see the cited references in each sub-section.

### 3.1. Generalized Linear Mixed Models

When considering theoretical statistical models, many issues should be evaluated, including (i) first, the distributional assumptions for the dependent variable and random errors; and (ii) second, the type of independent variables involved in the model. With this in mind, GLMMs could be viewed as an extension of Generalized Linear Models (GLMs) [12,13] where linear models are enlarged to consider the exponential family for the dependent variable distribution assumptions. At the same time, GLMMs also evaluate the inclusion in the model of random effects, and this feature extends this class of models with respect to mixed linear models [14]. Therefore, the main theoretical characteristics for GLMMs [15,16] can be summarized in two key-points: (i) the distributional assumption for the response variable *Y*; and (ii) the evaluation of fixed and random effects involved in the model as independent variables. Obviously, the GLMM theory and the advantages obtained by applying this class of models cannot be restricted to two key-points alone; in [17], GLMMs were applied to improve the semiconductor yield and to provide significant information when using a large dataset; however, these two key-points were essential for the theory applied in this study.

Let us consider a random variable *Y* with the expected value E(Y)=μ and variance $Var\left(Y\right)=\sigma^{2}$; we also defined the *i*-th realization of $Y_{i}$ as $y_{i}$. Moreover, each $Y_{i}$ was supposed to be distributed according to a density function belonging to the exponential family. The distribution for each $Y_{i}$ conditioned to the random effects γ can be expressed as

(2) $$\begin{array}{c} y_{i}|\gamma ∼\;ind.f_{Y_{i}|\gamma }\left(y_{i}|\gamma \right)\\ f_{Y_{i}|\gamma }\left(y_{i}|\gamma \right)=\exp\left[\frac{y_{i}\theta_{i}-b\left(\theta_{i}\right)}{a(\phi )}+c\left(y_{i},\phi \right)\right] \end{array}$$

where $\theta_{i}$ is the canonical parameter for each $Y_{i}$, ϕ is the scale parameter, and $b(\theta_{i})$ is a function for the mean and is equal to $\mu_{i}^{2}/2$ in the Gaussian case. The variance function $V\left(\mu_{i}\right)=\partial^{2}b\left(\theta_{i}\right)/\partial \theta_{i}^{2}$ expresses the relation between $y_{i}$ and $\mu_{i}$ and is one of the main elements for $var(y_{i})$ when considering the marginal variance in the presence of random effects [15]. These elements were differentiated according to the specific probability density function (p.d.f.) belonging to Formula (2). Moreover, the variance function played a relevant role in the 1990s when GLMs were introduced as alternative models for improving Taguchi’s two-step procedure [18] for modeling the location and dispersion of the response variable *Y*, also called the dual-response approach. Details of this well-known approach are presented in Section 3.3 below.

Given Formula (2), a GLMM can be defined with respect to a GLM model by adding random effects to the linear predictor $\eta_{i}=x_{i}^{{}^{\prime }}\beta$, where the distribution for each $Y_{i}$ is conditioned to the random effects γ. Therefore, a GLMM can be defined through the following three equations:

(3) $$\begin{array}{c} E\left(Y_{i}|\gamma \right)=g^{-1}\left(x_{i}^{\prime }\beta +z_{i}^{\prime }\gamma \right)=g^{-1}\left(\eta_{i}\right)=\mu_{i} \end{array}$$

(4) $$\begin{array}{c} Var(\gamma )=G; \end{array}$$

(5) $$\begin{array}{c} Var\left(Y|\gamma \right)=A^{1/2}BA^{1/2} \end{array}$$

where *g* is the link function that is equal to the identity function when Y|γ is assumed to be normally distributed; γ is the unknown vector of the coefficients for random effects; B=diag[1/a(ϕ)] if a diagonal structure is assumed for the variance-covariances (null covariances) of the residual error; *A* is a diagonal matrix formed by the variance function $V(\mu_{i})$. As in a linear mixed model [14], the array *G* is related to the variances and covariances of the random effects. Moreover, when considering the analysis of experimental variables in order to set a robust design, the inclusion of random effects allowed us to evaluate the effects on process variability due to noises that cannot be directly controlled, even though they are measurable. It must be noted that Formulas (3)–(5) fully define a general GLMM, namely, the model of Formula (3), the array *G* of variance-covariances for random effects (4), and lastly, the array defining the variance-covariances for the response variable *Y*, Formula (5).

### 3.2. The Split-Plot Design and Modeling

Split-plot designs were first defined in the 1950s [19]. Over the last two decades, following the seminal contributions by [3,20,21,22,23,24], split-plot design has received great attention as a valid experimental design in the technological field and for a robust design approach. In [3], this experimental design was developed by considering its specific framework in which the key aspect is the distinction between Whole-Plot (WP) and Sub-Plot (SP) factors, and three arrangements were suggested in order to consider the classification and environmental (noise) factors in an efficient setting. Moreover, the bi-randomization of the split-plot design may be useful to study noise and block variables as WP factors, also considered as random effects. Subsequently, the experimental variables, e.g., SP factors, are randomized within the WP units. Furthermore, the inclusion of experimental variables as SP factors guarantees more accurate estimations of these effects. Further theoretical developments have contributed towards expounding the relevance of the split-plot design. In [23], the equivalence between the Ordinary Least Squares (OLS) and Generalized Least Squares (GLS) estimation methods for fixed effects was established for cases where the split-plot design is planned according to a specific structure.

Next, we consider having WP factors as random variables and SP factors as fixed ones. This choice is strictly related to the case study illustrated below.

In general, a split-plot design is formed by two sets of factors in a bi-randomized structure: the set of Whole-Plot (WP) factors, $z=\{z_{1},...,z_{i},...,z_{I}\}$, and the set of Sub-Plot (SP) factors, $x\;=\;\{x_{1},...,x_{j},...,x_{J}\}$. Therefore, within each block (replicate) *k*, (k=1,...,K), for a balanced split-plot design, we have *n* runs, and consequently, the total number of trials is N=n*K.

For the sake of simplicity, let us start by considering a split-plot design with one WP factor ($z_{i}$) and one SP factor ($x_{j}$). Moreover, let us assume we have *K* blocks and that the WP factor is involved in the model as a random variable while the SP factor is included as a fixed effect. Therefore, by also considering the general formulation (3), the corresponding GLMM for a single observation can be expressed as

(6) $$\begin{array}{c} \eta_{ijk}=\mu +\xi_{k}+\gamma_{i}+\zeta_{ik}+\beta_{j}\\ \gamma_{i}\;iid∼N\left(0,\sigma_{\gamma }^{2}\right)\\ \xi_{k}\;iid∼N\left(0,\sigma_{b}^{2}\right)\\ y_{ijk}=\mu +\xi_{k}+\gamma_{i}+\zeta_{ik}+\beta_{j}+\epsilon_{ijk}\\ \left(y_{ijk}|\gamma_{i},\xi_{k}\right)∼N\left(\mu_{ijk},\sigma_{SP}^{2}\right)\\ \zeta_{ik}∼N\left(0,\sigma_{WP}^{2}\right)\\ \epsilon_{ijk}∼N\left(0,\sigma_{SP}^{2}\right)\\ link:\eta_{ijk}=\mu_{ijk} \end{array}$$

where $\eta_{ijk}$ is the linear predictor; $\xi_{k}$ is the replicate (block) effect; and $\zeta_{ik}$ and $\epsilon_{ijk}$ are the WP and SP errors respectively. Please note that $\zeta_{ik}={\left(\gamma \xi \right)}_{ik}$, e.g., the WP error, is estimated through the first interaction term between replicates and the WP factor. The two unknown coefficients $\gamma_{i}$ and $\beta_{j}$, which are random and fixed respectively, relate to the two effects for the WP and SP factors. It must be noted that $var\left(y_{ijk}\right)=\sigma_{WP}^{2}+\sigma_{SP}^{2}$. Furthermore, by considering the distributional assumption of Formula (6), we can define the array for the variance-covariances of the response variable as follows:

$$V=ZGZ^{\prime }+R$$

where *R* is the array for the variance-covariances of the residual error if the Normal distribution is hypothesized. In general, a diagonal structure can be assumed for the two arrays, *G* and *R*, i.e., explicitly referring to a variance component structure for both in which all the covariances are assumed to be null. Nevertheless, for a split-plot design/structure, *G* and/or *R* arrays can be structured differently, e.g., a diagonal structure is often misleading. In fact, no null covariances can occur when considering the residual error and/or random effects conditioned to blocks and/or categorical WP factors, such as a classification variable. In this case, we may assume that a covariance is present among observations belonging to the same Whole-Unit (WU), formed by the level combination of WP factors in the *k* block; otherwise the covariance is null.

Therefore, we may consider a partition for array *G* by discriminating between $G_{ii}$ and $G_{ii^{\prime }}$: $G_{ii}$ is the part of *G* related to the variance for random effects; $G_{ii^{\prime }}$ is related to the covariances between random effects. By considering the model in Formula (6), we can express *V* as follows:

(7) $$\begin{array}{c} V=ZGZ^{\prime }+R \end{array}$$

(8) $$\begin{array}{c} V=\sum_{i}Z_{i}G_{ii}Z_{i}^{{}^{\prime }}+\sum_{i}\sum_{i^{\prime }}Z_{i}G_{ii^{\prime }}Z_{i}^{{}^{\prime }}+R;i\neq i^{\prime }. \end{array}$$

The general structure for V (Formulas (7) and (8)) may be better defined for a split-plot design when considering a Compound Symmetry (CS) structure.

Regarding the CS structure, by denoting the array $\left[1_{n}^{{}^{\prime }}\times 1_{n}\right]$ with *J* for each block/replicate (*n* dimension), the array *G* becomes

(9) $$G=\sigma^{2}I_{n\times n}+\sigma_{CS}J_{n\times n}.$$

In Formula (9), the variances of the random effects are formed by $\sigma^{2}+\sigma_{CS};\sigma_{ii}^{2}=\sigma^{2}\forall i$, and covariances are assumed to be constant and are equal to $\sigma_{CS}$. Moreover, the CS structure for the *G* array allows the no-null covariances to be evaluated among units within the same WU and the same replicate, while variances are related to the random effects, such as γ and the replicate effect of model (6). The variance component of the WP error is then given by the interaction between the random factor $z_{i}$ and the replicates, while the array *R* is related to the variance-covariances for the SP error (residual). In the case-study we evaluated a CS structure for *G* and a diagonal structure for *R*.

### 3.3. The Dual-Response Generalized Linear Mixed Models Approach

Over the past few decades, response surface methodology (RSM) and GLMs have been widely and permanently introduced to define alternative methods to Taguchi’s two-step procedure an the robust design approach [18,25], where two statistical models for the response variable, e.g., location and dispersion models, are defined. RSM has been extensively applied in order to improve Taguchi’s parameter design, especially considering the concept of a robust design and the two-step procedure (dual-response modeling approach). The dual-response approach was initially addressed with consideration of both RSM [25] and the class of GLMs [18]. Nevertheless, the sequential nature of the RSM, that is, the experimental design and statistical model, certainly represents an advantage for the application of this methodology [26]. The combined array is a valid alternative to improve both the two-step procedure and the robust design [27]. On the other hand, GLMs confirm their important role in this field, and they have been integrated with the local optimal experimental design theory [28]. In this sense, GLMMs represent a further improvement due to the peculiarities belonging to this class of models that extend GLMs towards the inclusion of random effects; moreover, also with respect to RSM, they could be more suitable for specific experimental designs, such as the split-plot, when considering the split-plot structure and the GLMM features illustrated in Section 3.1.

Therefore, in this section, the proposal of a dual-response approach arises from the need to account for the residual variability of the response by modeling the residuals of the location model. As a result, we have (i) a main (location) split-plot model including random as well as fixed effects, in which residual weighting is involved in order to account for overdispersion; (ii) a dispersion model, in which the dependent variable is defined by the residuals obtained as a first step, through the estimation of the location model by considering the homoscedasticity assumption. It must be noted that the estimated residual values obtained from the fitting of the dispersion model are called “residual weighting”, and they are used as weights for the subsequent estimated location model in order to evaluate the overdispersion.

Starting from the main split-plot model (Formula (6)), in general, the dispersion model can be defined according to the following formula, where υ is the expected value for the residuals:

(10) $$\begin{array}{c} v_{ijk}=\upsilon +\beta_{j}+\varepsilon_{ijk} \end{array}$$

(11) $$\begin{array}{c} \left(v_{ijk}|\gamma_{i},\xi_{k}\right)∼Q\left(\upsilon ,\upsilon^{2}\right)\\ \varepsilon_{ijk}∼N\left(0,\sigma^{2}\right)\\ link:\eta_{ijk}=log\left(\upsilon_{ijk}\right) \end{array}$$

and the variance function $V\left(\mu \right)=V\left(\upsilon \right)=\upsilon^{2}$. This dual-response modeling, e.g., location and dispersion models, is iteratively performed. The residual values of the dispersion model are used as weights, and the main split-plot model (Formula (6)) is newly fitted to obtain a better fitting of the model for coefficient estimates, predicted values, diagnostic measures, and analyses. The better fitting is obviously tested through diagnostics, as shown in the following case-study.

Moreover, the dispersion model, Formula (10), is fitted through a Quasi-Likelihood (QL) estimation method, e.g., by only specifying the mean and the variance function (first two moments) for the dependent variable [13,29,30]. Therefore, the application of the QL estimation method allows for obtaining a better fitting of the weighted location model by involving overdispersion and avoiding the specification of the probability distribution function (p.d.f.) for the dispersion model.

Regarding the kind of residuals chosen for weighting, we applied the Pearson residuals here, e.g., each residual ($r_{y}=y-g^{-1}\left(\hat{\eta }\right))$ was standardized by the square-root of the estimated conditional variance, calculated for each observation:

(12) $$r_{y}^{p}=\frac{r_{y}}{\sqrt{\hat{Var}\left(y|\gamma \right)}}.$$

## 4. The Case Study: Description of the Split-Plot Planning

As mentioned above, the theoretical concepts of this study were applied to optimize gas sensing materials conditioned by temperature values. The response variable is illustrated in Formula (1), Section 2.

We decided to plan a split-plot design (similar to [5]), as it is particularly valid in this situation where each chamber may naturally be involved in the study as a replicate; moreover, the bi-randomization of the split-plot allowed the entire experimentation to be conducted more effectively, as illustrated below. In addition, the decision to apply GLMMs jointly with a dual-response approach has two aims: (i) in order to define one model only for both Target gases implies the need to account for location and dispersion effects, in which the dispersion effects are studied in a distinct model and predicted residuals are used for studying heteroscedasticity. This enabled us to better achieve the two different targets (even though both maximized the response values) at different temperatures, and to minimize the process variability. (ii) The application of GLMMs has proven to be a flexible tool for studying random effects together with the bi-randomized structure of the split-plot design, and also allows for achieving better model and diagnostic results through a QL approach for the dispersion model.

The first and most important step that we followed when planning this split-plot design was to consider all of the sources of variability involved in the process. For example, we distinguished between two temperatures: (i) the temperature of the measurement chamber; and (ii) the temperature measured by the Resistive Temperature Device (RTD) on the sensor, also called the working temperature. The latter played a relevant role in the final experimental design, while the former was evaluated as an external variable, e.g., it was not involved in the experimental design.

More specifically, we planned a split-plot design with the block variable, target gas (gas), at two levels: two gases, NO${}_{2}$ and CO, coded as gas1 and gas2 in Table 2, respectively. For each target gas, a total of six chambers were studied; eight sensors were studied within each chamber, each sensor relating to a gas sensing material. Each chamber studied for each target gas was measured at a specified gas concentration level and replicated at two humidity levels. Therefore, after being conditioned by the type of target gas, three chambers were evaluated for each level of gas concentration; at each gas concentration level, two levels of humidity (dry/wet) were investigated. Thus, we processed six chambers for each target gas at three levels of gas concentration studied at two levels of humidity. A WU was formed by the combination of the gas concentration and humidity level; therefore, we had 6 WUs for each gas. Furthermore, the gas concentration was included in the experimental design as a WP factor. We considered three different concentration values ranging from 70 to 285 ppm for CO by referring to the lowest limit for the activation of consumer alarms (e.g., 70 ppm for UL2034, standard and multiple station carbon monoxide) and the early health effects, e.g., headache and nausea after 23 h of exposure to over 200 ppm of CO. Regarding NO${}_{2}$, we chose a range equal to [6–16] ppm to include a wide variety of commercial and industrial applications, such as diesel exhaust in parking structures, tunnels, and ventilation systems. Moreover, humidity is an environmental variable that was also included as a WP factor at two levels (wet and dry), corresponding to 0% and 35%, which are the minimum and maximum level values of humidity for detecting dangerous gases, such as NO${}_{2}$ and CO, when the sensor is installed; the humidity is induced through an a priori setting of the thermostatic bath. The evaluation of humidity in the experiment was related to the need to confirm the negligible effect of this factor at low values. In addition, as stated in literature [31], the presence of humidity does not have much of an effect on the conductivity in air. The resistance measured in air and humid air is very similar, and the difference is smaller than the measurement uncertainty. Nevertheless, the influence of humidity also depends on the type of sensing materials, which are, at times, very sensitive to humidity and capable of generating drifts or false positives in the response values of the electrical resistance. To this end, we chose to include humidity at low levels in order to obtain confirmation that there was (i) no effect on the sensing film; and (ii) no sensitivity to the humidity of the sensing films studied here.

As also stated in Section 3.2, we decided to study the WP factors, e.g., gas concentration and humidity as random effects and the SP factors, e.g., material type and working temperature, as fixed effects. This choice was related to the warm-map ([2]), e.g., the set-up (Section 2, Figure 1 parts a,b) of the gas concentration and humidity (Figure 1 part c) in order to control the presence of noise variables that could alter the levels of the WP factors. In fact, the sensors were stabilized in synthetic air for 30 min (long enough to simulate the operating conditions in which sensors are installed) before being exposed to the target gas, which was the set-up of the measurement system involving the SP factors (Figure 1 parts q,r). In addition, even though the gas concentration is a process variable, it is difficult to control with any degree of accuracy. In [32], the resolution of a similar problem was conducted analogously, albeit in a different context.

The gas sensing materials (eight types of perovskites) and the working temperature were considered as SP factors. The material type was evaluated as a categorical variable at eight levels, while the temperature was studied by considering a different range of values (${}^{\circ }$C) per target gas. In order to better evaluate the working temperature, we studied four temperature intervals for each target gas. The two SP factors were the main process variables and they played a key role in process optimization.

**Figure 2:**
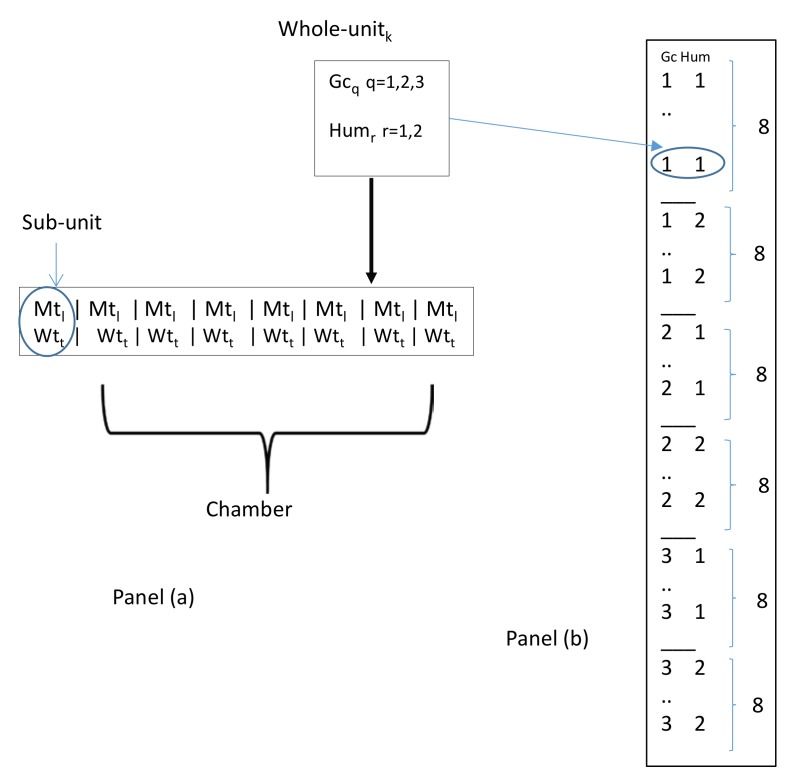
Panel (**a**) The split-plot structure for each gas with a general Whole-Unit (WU) and the related Sub-Unit (SU). (*l* = 1, ..., 8; *t* = 1, ..., 4). Panel (**b**) shows the WP structure with the 6 WUs, with each WU split into 8 SUs. Gc: gas concentration; Hum: humidity; Wt: working temperature; Mt: material type.

Therefore, for each target gas, we applied a split-plot design with two WP factors (humidity and gas concentration) and two SP factors (material type and working temperature-wt). In Figure 2, we illustrate the split-plot structure for each gas.

It must be noted that within each WU, the eight sensor materials were randomized at a pre-specified working temperature level and then all put together in the chamber set with a specific combination of gas concentration and humidity levels. The total number of experimental observations was 96: therefore, we had 48 observations for each target gas.

The environmental temperature for each chamber was measured during the experimentation step and considered as a random variable in the statistical model; however, it was not relevant for the whole process, even as an external variable. Other sources of variability were considered in the planning and subsequent measurement process: (i) the differences in heating during the manufacturing of the sensors for each type of perovskite, which was included in the analysis through the material type; and (ii) the gas-flow, which was kept constant at 300 mL/min. Table 2 contains detailed descriptions of the experimental variables.

## 5. The Case Study: Modeling Results

This section illustrates the results obtained by applying the proposed dual-response modeling approach (Section 3) to our case study. Next, we illustrate the results for the main split-plot model (Table 3, Table 4 and Table 5) and for the related dispersion model (Table 6 and Table 7) applied at iteration number 2. Given the assumed hypotheses for the main GLMM model, the related model for dispersion was fitted with a QL approach, in which $V\left(\mu \right)=V\left(\upsilon \right)=\upsilon^{2}$. Therefore, iteration number 2 (Section 3.3) means that the dual-response modeling approach has been iterated twice. The location (main) model, with Identity link and Normal distribution, was weighted with the absolute value of residuals obtained by fitting the dispersion model during the previous step.

The GLMM applied for the split-plot design (Section 2 and Section 4), and expressed for a single observation *y*, was the following:

(13) $$\begin{array}{c} y=\mu +\sum_{q(s)=1}^{3}\gamma_{Gc_{q(g_{s})}}*z_{Gc}+\sum_{l=1}^{l-1}\beta_{Mt_{l}}*x_{Mt_{l}}+\\ +\sum_{s=1}^{s-1}\beta_{g_{s}}*x_{g_{s}}+\beta_{T}*x_{T}+\sum_{q(s)=1}^{3}\epsilon_{q(g_{s})}\\ q=1,\;\cdots ,\;3;l=1,\;\cdots ,\;8;s=1,2. \end{array}$$

It must be noted that ${}_{Gc_{q(g_{s})}}$ is the general random coefficient (q=1,2,3) for the gas concentration per target gas (s=1,2); $\beta_{Mt_{l}};l=1,\;\cdots ,\;8$ are the parameters related to the material type factor; $\beta_{g_{s}}$ and $\beta_{T}$ are the coefficients for the target gas (s=1,2) and working temperature, respectively. The six coefficients $\epsilon_{q(g_{s})}$ are the residual errors estimated for each gas concentration level conditioned per target gas. It can also be observed how the first error term is formed by the interactions between the replicate effect and the WP factors; the WP effects were tested against this error component. The second error term was the residual error of the model.

The Kenward–Roger method was chosen for the computation of the degrees of freedom for the denominator in hypothesis tests for fixed effects. The estimation of the fixed part of model (13) was obtained via the Generalized Least Squares (GLS) method while the estimation of variance components was carried out using the Restricted Maximum Likelihood (REML) method. Moreover, the matrix *G* of variance-covariance for random effects (Table 4) had a compound symmetry structure with ${\hat{\sigma }}^{2}=6.97$ and ${\hat{\sigma }}_{CS}=-3.57$, Formula (9). The error component was estimated by considering the gas concentration within each target gas. *R* was a diagonal matrix formed by a six block array $R_{q(g_{s})}$; each error variance component was the diagonal element of the corresponding 6 blocks with dimensions [16×16], as shown in Table 5. At the beginning, when applying the main model for the split-plot design, two first order interactions were investigated: the material type and working temperature and the material type and target gas; both were significant when considering the global effect (Sum of Squares of Type III). Nevertheless, the interaction with temperature was only significant for one of the seven estimated coefficients, while the second interaction, relating to the target gas, did not allow for improving residuals and predicted values, even though the comparison was made with subtle distinctions.

Moreover, the humidity factor, involved in the experimental design as a WP factor, did not give significant results and was not included in the final reported model. This result confirms that low humidity values have no influence on either the sensor packaging or the type of sensing film studied here.

The dispersion model, shown in Formulas (10) and (11), used for weighting the main model (13) to account for overdispersion, is briefly illustrated. The model expression, written for a single observation, where the response variable *y* is denoted here by *v*, is the following:

(14) $$\begin{array}{c} v=\upsilon +\sum_{l=1}^{l-1}\beta_{Mt_{l}}*x_{Mt_{l}}+\beta_{T}*x_{T}+\sum_{s=1}^{s-1}\beta_{g_{s}}*x_{g_{s}}+\sum_{s=1}^{s-1}\varepsilon_{g_{s}}\\ l=1,\;\cdots ,\;8;s=1,2. \end{array}$$

Note that $\beta_{Mt_{l}};l=1,\;\cdots ,\;8$ are the parameters related to the material type factor, estimated here with respect to the residuals; $\beta_{T}$ is the coefficient for the working temperature, while $\beta_{g_{s}}$ are related to the estimates for the target gas effects. The two coefficients $\varepsilon_{g_{s}}$ are the two residual errors estimated for each target gas.

Table 6 and Table 7 contain the estimated coefficients for the fixed effects and the Residual Pseudo-Likelihood (RSPL) estimates for the residual error, respectively. The choice to perform error estimates with the RSPL method was based on the assurance that the residual method accounts for the fixed effects in the construction of the objective function. It must be noted that error estimates are the two scale parameters for each target gas, which show a slightly different performance in the error components. The results for fixed effects (Table 6) were as expected, e.g., the coefficients were less significant, often negligible, especially for the material types. Finally, an advantage of this analysis is the possibility of applying only one model for both target gases, and then optimizing both target gases on the basis of the same estimated modelling approach. This choice is undoubtedly interesting from an engineering point of view if we also evaluate the optimal predicted values illustrated in the next section. Furthermore, the statistical analysis assessed the different behaviors of the response (Formula (1)) for each target gas.

**Figure 3:**
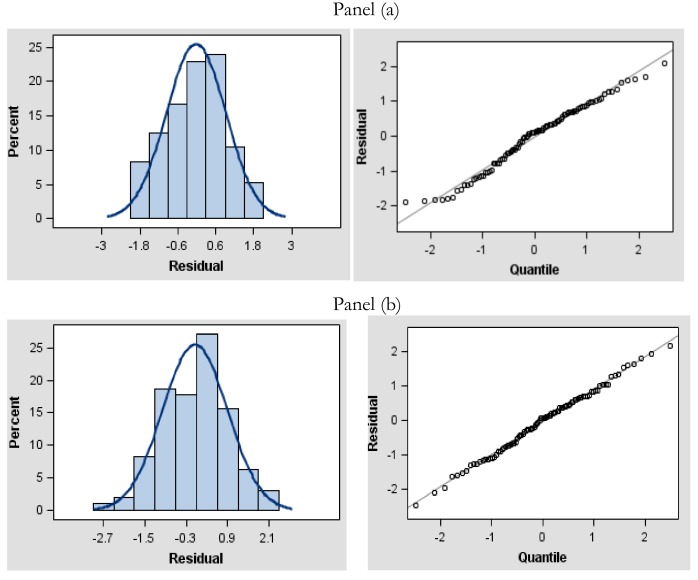
Iteration number 1 (panel (**a**)) and iteration number 2 (panel (**b**))—Pearson residual histogram with Normal probability density function (p.d.f.) and Q-Q plot for the main weighted split-plot model.

Undoubtedly, given the distributional and link assumptions for the main model, a natural consequence has been the investigation of a dispersion GLMM with Gamma as the distributional assumption; however, this a priori hypothesis has not been confirmed by analysis, as also confirmed by the estimated scale parameter. Furthermore, the improvement achieved by the estimation through a QL approach may be observed from the diagnostic analysis of Pearson residuals of the main model fitted at iteration number 2, as also illustrated by Figure 3, relating to the first iteration (panel a) and second iteration (panel b) for the main weighted model.

The diagnostic statistics (-2 Residual Log-Likelihood; Akaike Information Criterion—AIC, Bayesian information criterion—BIC indexes) showed notable improvements for the dispersion model when passing from iteration number 1 to number 2; the same cannot be affirmed for the main model (13), where the fit statistics did not show any real changes. Nevertheless, the ratio between the Generalized Chi-square and degree of freedom was always very close to one.

The analysis of the case study was carried out by applying the GLIMMIX procedure (SAS Software; version 9.2, SAS Institute Inc., Cary, NC, USA).

## 6. Optimized Gas Sensing Materials: Discussion

Table 8 shows the optimal solutions obtained as predicted values of the weighted main model (13) fitted at iteration number 2. The predicted values were calculated for all combinations of target gas and material type. Within the set of predicted values for each combination, the best solution was chosen according to the maximum response, minimum working temperature, and the estimated Pearson residual (Formula (12)), the latter being considered preferable when at low or minimum values. In general, the results achieved from this case study are a great improvement on the previous results obtained by [4,5], also due to the application of the dual response approach with GLMMs for the two target gases together. This modeling choice also implies that optimization can be carried out without involving analytical procedures and optimization algorithms, thus saving an additional computational burden; the optimized response values obtained fully satisfy the engineering requirements conditioned to the working temperature. It is relevant to note that if the gas concentration increases, this implies an increment in the response up to a saturation level. Therefore, when considering the results obtained, we mostly pay attention to achieving the highest response values with a parity of gas concentration.

In particular, in Table 8, it can be observed that the solutions are acceptable from an engineering point of view. The responses are satisfactory for NO${}_{2}$ sensors, because they also combine an efficient response with a low working temperature. The material type Mt4, Y${}_{1.1}$CoO${}_{3.15}$ achieved the best solution with the response value optimized at -22.20% with a working temperature of 194.97 ${}^{\circ }$C. In regard to Mt6, YCo${}_{0.9}$O${}_{2.85}$, the best response value achieved was equal to -18.12% with a temperature equal to 194.60 ${}^{\circ }$C. The low gas concentration for NO${}_{2}$, equal to 6.33 ppm, made Mt6 suitable for the detection of gas1. Undoubtedly, compared to Mt4, Mt2 and Mt3 achieved good response values with lower working temperatures and the same value of gas concentration. However, we consider Mt4 the best solution (*ceteris paribus* for gas concentration) as it gave the maximum response with a working temperature difference of ≈+29 ${}^{\circ }$C, which is still an acceptable value in operating terms.

Regarding gas2 (Table 8) (CO), the materials met all the stringent engineering requirements. Excellent results were achieved for Mt7. In particular, $YCoO_{3}$ gave satisfactory solutions with the response value optimized at +17.49% with a temperature equal to 284.67 ${}^{\circ }$C. The material type Mt8 could be a valid alternative to Mt7, because it achieves a response value equal to 9.56% at a low gas concentration value of 71.0 ppm; however, when considering higher levels of gas concentration, this material did not give satisfactory results.

Mt2 (YCo${}_{0.9}$Pd${}_{0.05}$O${}_{3}$) is useful and feasible for the detection of both gases with an optimal response equal to +12.44% and a relatively low working temperature of 264.90 ${}^{\circ }$C for CO and a large response equal to −15.55% at a temperature of 164.99 ${}^{\circ }$C for NO${}_{2}$.

Nonetheless, the implementation reserved for ambient air pollution monitoring and industrial application requires further study because the MOX sensor that works at different temperatures, actually shows different response speeds and selectivity degrees of gases [33].

## 7. Concluding Remarks

This paper dealt with split-plot planning and dual-response modeling applied to a case study on new gas sensing materials in a robust process optimization context where random and fixed effects were considered. The approach was suggested in order to improve the applications of experimental designs in the technological field by means of methodological as well as applied perspectives. The final results confirmed the validity of the suggested approach, by also evaluating the modeling checks with diagnostics. When compared with previous studies, this research showed a better performance following the theoretical improvements—the planned design evaluates six chambers for each target gas and two chambers for each gas concentration value at two humidity levels, the dual-response approach allows the location model to be weighted in order to account for overdispersion, and a compound symmetry structure is studied for the array of variance-covariances of random effects. Moreover, the very positive model results allow satisfactorily high response values to be obtained for all sensing films with acceptable working temperature values, without having to perform an analytical approach for each sensor (categorical variable).

From an engineering perspective, our results confirm the possibility of applying experimental designs and statistical analyses in order to reduce the calibration time and characterize novel sensor materials through a systematic selection of tests aimed at gathering the maximum amount of information possible with the least number of observations. In this way, the cost of gas sensor characterization is minimized in terms of man-hours and budget, while the quantitative results and learning are maximized. Moreover, the proposed materials can be used for the in situ detection of combustion of byproducts (CO and NO${}_{2}$) in industrial applications, and also to monitor the environmental pollution in real-time. The research on these materials for studying and characterizing gas sensitivity and selectivity based on the field of application together with their stability and the reliability is ongoing. 

## Figures and Tables

**Table 1:** Gas-sensing materials: chemical compositions and symbols.

Chemical Composition	Symbol
YCo${}_{0.9}$Pd${}_{0.1}$O${}_{3}$	Mt1
YCo${}_{0.95}$Pd${}_{0.05}$O${}_{3}$	Mt2
Y${}_{0.95}$CoPd${}_{0.05}$O${}_{3}$	Mt3
Y${}_{1.1}$CoO${}_{3.15}$	Mt4
YCoO${}_{3}$	Mt5
YCo${}_{0.9}$O${}_{2.85}$	Mt6
YCoO${}_{3}$ + 1%Pd	Mt7
YCo${}_{0.9}$O${}_{2.85}$ + 1%Pd	Mt8

**Table 2:** Experimental variables.

Factor	Name	Symbol	Range	Target Gas	Part in Figure 1
WP	humidity (%)	$z_{H}$	[0–35]	gas1,2	bubbler, part c
WP	gas concentration (ppm)	$z_{Gc}$	[6–16]	gas1	mass flowmeters, part b
WP	gas concentration (ppm)	$z_{Gc}$	[70–285]	gas2	mass flowmeters, part b
SP	material type	$x_{Mt_{l}}$	see Table 1	gas1,2	screen-printed sensing material, part r
SP	working temp. (${}^{\circ }$C)	$x_{T}$	[165–210]	gas1	screen-printed sensing material, part r
SP	working temp. (${}^{\circ }$C)	$x_{T}$	[240–310]	gas2	temperature sensor, part q

WP: Whole-Plot; SP: Sub-Plot.

**Table 3:** Generalized Least Squares (GLS) estimates for the fixed effects of the model (13).

Coefficient	Estimate	S.E.	*p*-Value
μ	10.0541	1.6690	0.0001
$\beta_{T}$	−2.3349	0.7101	0.0031
$\beta_{Mt_{1}}$	−2.8207	1.5007	0.0720
$\beta_{Mt_{2}}$	0.2056	1.5260	0.8939
$\beta_{Mt_{3}}$	2.4623	1.4992	0.1140
$\beta_{Mt_{4}}$	−5.0430	1.5526	0.0032
$\beta_{Mt_{5}}$	0.0356	1.5040	0.9813
$\beta_{Mt_{6}}$	−1.8843	1.5079	0.2234
$\beta_{Mt_{7}}$	6.1855	1.5169	0.0004
$\beta_{Mt_{8}}$	0	.	.
$\beta_{g_{1}}$	−28.6938	2.6548	0.0001
$\beta_{g_{2}}$	0	.	.

**Table 4:** Estimates for the random effects of the model (13).

Coeff. (Effect)	Estimate	S.E.	*p*-Value
$\gamma_{Gc_{1(g_{1})}}$	−0.9616	1.9643	0.6765
$\gamma_{Gc_{2(g_{1})}}$	0.7538	1.9504	0.7433
$\gamma_{Gc_{3(g_{1})}}$	0.1521	1.9443	0.9457
$\gamma_{Gc_{1(g_{2})}}$	0.0016	1.8464	0.9994
$\gamma_{Gc_{2(g_{2})}}$	−0.5470	1.8345	0.7846
$\gamma_{Gc_{3(g_{2})}}$	1.9784	0.5811	0.0082

**Table 5:** Restricted Maximum Likelihood (REML) estimates for the residual errors of the model (13).

Coeff.	est.	S.E.
$\epsilon_{1(g_{1})}$	152.35	57.14
$\epsilon_{2(g_{1})}$	294.78	108.08
$\epsilon_{3(g_{1})}$	19.15	6.94
$\epsilon_{1(g_{2})}$	30.81	11.73
$\epsilon_{2(g_{2})}$	2.62	1.15
$\epsilon_{3(g_{2})}$	3.08	1.34

**Table 6:** GLS estimates for the fixed effects of the dispersion model.

Coefficient	Estimate	S.E.	*p*-Value
υ	2.4315	0.1825	0.0001
$\beta_{T}$	−0.1419	0.1139	0.2175
$\beta_{Mt_{1}}$	0.04031	0.1403	0.7751
$\beta_{Mt_{2}}$	0.03786	0.1407	0.7890
$\beta_{Mt_{3}}$	0.0758	0.1435	0.5998
$\beta_{Mt_{4}}$	0.1165	0.1404	0.4109
$\beta_{Mt_{5}}$	0.1239	0.1499	0.4128
$\beta_{Mt_{6}}$	0.1078	0.1402	0.4458
$\beta_{Mt_{7}}$	0.0466	0.1424	0.7451
$\beta_{Mt_{8}}$	0	.	.
$\beta_{g_{1}}$	2.6498	0.2440	0.0001
$\beta_{g_{2}}$	0	.	.

**Table 7:** Residual Pseudo-Likelihood estimates for errors of dispersion model.

Coeff.	est.	S.E.
$\varepsilon_{g_{1}}$	0.0627	0.0140
$\varepsilon_{g_{2}}$	0.8238	0.1707

**Table 8:** Optimal values and $r_{y}^{p}$ obtained through the fitted main model (iteration no. 2) for gas NO${}_{2}$ and gas CO.

Optimal Value	Mt1	Mt2	Mt3	Mt4	Mt5	Mt6	Mt7	Mt8
				Target gas = $NO_{2}$				
Response	−19.76	−15.55	−13.29	−22.20	−18.30	−18.12	−11.67	−16.94
Working Temperature	179.90	164.99	165.00	194.97	209.90	194.60	209.90	179.90
Gas Concentration	11.09	15.83	15.83	15.83	11.09	6.33	15.83	11.09
$r_{y}^{p}$	0.59	0.35	0.06	−0.14	0.21	−0.70	0.09	0.12
				Target gas = CO				
Response	7.33	12.44	15.86	4.22	9.29	11.52	17.49	9.56
Working Temperature	309.39	264.90	239.90	239.70	239.90	239.80	284.67	239.71
Gas Concentration	284.0	284.0	284.0	142.0	142.0	284.0	284.0	71.0
$r_{y}^{p}$	−0.11	0.07	0.42	0.13	−0.32	0.06	0.22	−0.25
